# OxDBase: a database of oxygenases involved in biodegradation

**DOI:** 10.1186/1756-0500-2-67

**Published:** 2009-04-30

**Authors:** Pankaj K Arora, Manish Kumar, Archana Chauhan, Gajendra PS Raghava, Rakesh K Jain

**Affiliations:** 1 Environmental Biotechnology, Institute of Microbial Technology, Sector 39-A, Chandigarh-160036, India; 2 Bioinformatics Centre, Institute of Microbial Technology, Sector 39-A, Chandigarh-160036, India

## Abstract

**Background:**

Oxygenases belong to the oxidoreductive group of enzymes (E.C. Class 1), which oxidize the substrates by transferring oxygen from molecular oxygen (O_2_) and utilize FAD/NADH/NADPH as the co-substrate. Oxygenases can further be grouped into two categories i.e. monooxygenases and dioxygenases on the basis of number of oxygen atoms used for oxidation. They play a key role in the metabolism of organic compounds by increasing their reactivity or water solubility or bringing about cleavage of the aromatic ring.

**Findings:**

We compiled a database of biodegradative oxygenases (OxDBase) which provides a compilation of the oxygenase data as sourced from primary literature in the form of web accessible database. There are two separate search engines for searching into the database i.e. mono and dioxygenases database respectively. Each enzyme entry contains its common name and synonym, reaction in which enzyme is involved, family and subfamily, structure and gene link and literature citation. The entries are also linked to several external database including BRENDA, KEGG, ENZYME and UM-BBD providing wide background information. At present the database contains information of over 235 oxygenases including both dioxygenases and monooxygenases. This database is freely available online at .

**Conclusion:**

OxDBase is the first database that is dedicated only to oxygenases and provides comprehensive information about them. Due to the importance of the oxygenases in chemical synthesis of drug intermediates and oxidation of xenobiotic compounds, OxDBase database would be very useful tool in the field of synthetic chemistry as well as bioremediation.

## Background

**Figure 1:**
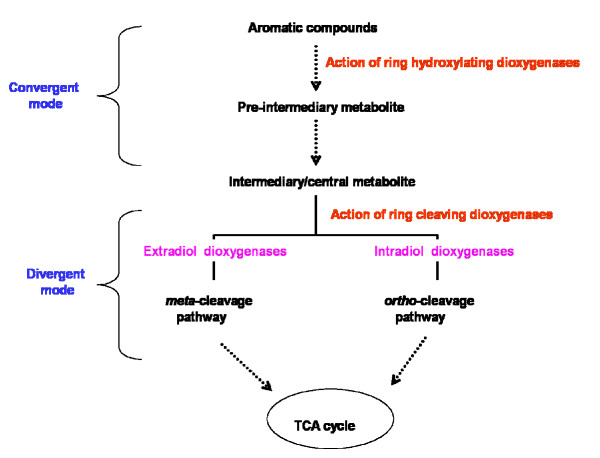
**Schematic diagram showing the role of aromatic dioxygenases in the bacterial degradation of aromatic compounds (Adapted from Khajamohiddin *et al*., 2008)**.

**Figure 2:**
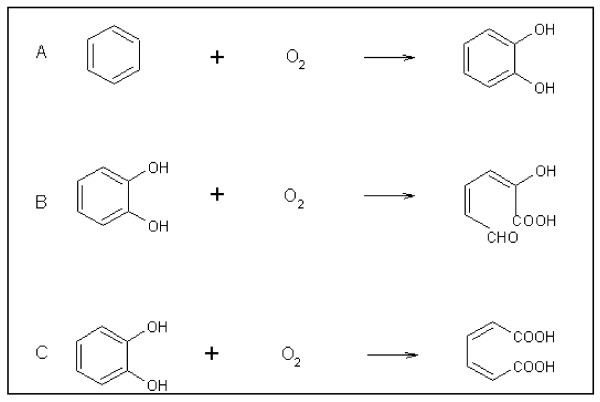
**Figure illustrating the mechanism of action of aromatic dioxygenases**. A) aromatic ring hydroxylating dioxygenase; B) extadiol ring cleavage dioxygenase; and C) intradiol ring cleavage dioxygenase.

In the last few decades, extensive urbanization and rapid industrialization has resulted in the addition of a large number of xenobiotic compounds into the environment. The chemical properties and quantities of the xenobiotic compounds determine their toxicity and persistence in the environment. Organic (aromatic/non-aromatic) compounds constitute a major group of environmental pollutants [1]. These compounds are highly persistent in the environment due to their thermodynamic stability [2]. Many of these compounds have been reported to be toxic to the living organisms [3]. Increased public awareness about the hazards and toxicity of these compounds has encouraged the development of technologies for their remediation. Bioremediation, which utilizes the microbial metabolic potential of the degrading microorganisms, has come up as an efficient and cost-effective means of large scale removal of these compounds in comparison to the physico-chemical means of bioremediation. A number of bacteria that can degrade a variety of aromatic compounds have been identified and the pathways involved in the degradation have been extensively characterized [3,4]. Based on the complexity of the degradation pathways, the phenomenon of biodegradation is categorized into two types: convergent and divergent modes of degradation (Fig. 1). In the convergent mode, structurally diverse aromatic compounds are converted to one of a few aromatic ring cleavage substrates such as catechol, gent sate, protocatechuate and their derivatives [5]. Peripheral enzymes, particularly oxygenases and dehydrogenases, were found to transform structurally diverse substrates into one of these central intermediates by bringing about the hydroxylation of the aromatic nucleus (Fig. 2A), and hence it is thought that bacteria have developed these enzymes to extend their substrate range [5]. There are a number of benefits of channeling diverse compounds into a few central aromatic ring cleavage substrates; the foremost among these being reduction of genetic load and simplification of regulatory circuits. Further, the centralized degradation pathways mean synthesis of fewer degradative enzymes requiring less metabolic energy. This is clearly a major advantage to soil microbes which often find themselves in unfavorable environments containing low concentrations of carbon sources suitable for growth [6]. However, further conversion of these intermediates into tricarboxylic acid (TCA) cycle intermediates was found to be highly diverged (divergent mode) (Fig. 1). In this divergent mode, a metal-dependent dioxygenase channels these dihydroxylated intermediates into one of the two possible pathways: the *meta*-cleavage pathway or the *ortho*-cleavage pathway [7-9] (Fig. 1). The substrate specificity of these metal-dependent dioxygenases has been found to play a key role in the overall determination of pathway selection [5] and the dioxygenases have been grouped into two classes namely extradiol and intradiol dioxygenases [7]. Extradiol dioxygenases have nonheme iron (II) at their active site and catalyze ring cleavage at the carbon-carbon (C-C) bond adjacent to the vicinal hydroxyl groups (*meta*-cleavage) (Fig. 2B) whereas intradiol dioxygenases have non-heme iron (III) in their active site and catalyze ring cleavage at the C-C bond between the vicinal hydroxyl groups (*ortho*-cleavage) (Fig. 2C). Extradiol dioxygenases channel substrates into a *meta*-pathway whereas intradiol dioxygenases channel these substrates into an *ortho*-pathway. Similarly, monoxygenases catalyze the transfer of one atom of molecular oxygen to the organic compound with other being reduced by electrons from cofactors to yield water thereby increasing their reactivity and water solubility.

Oxygenases are one of the key enzymes that play a central role in the degradation/detoxification of compounds. Without the activity of these oxygenases the mineralization of these xenobiotic compounds is not possible. Despite the fact that the oxygenases play such a crucial role, limited information is available with respect to these enzymes. None of the existing databases provide complete and/or comparative information on all the oxygenases known till date. Recent genomic, kinetics and crystallographic studies on oxygenases have increased our understanding of the distribution, evolution and mechanism of these enzymes [10]. Studies on oxygenases have also shown that extradiol dioxygenases are also involved in the biosynthesis of a variety of biologically active compounds e.g. lincomycin [11]. Keeping above in mind we have developed a database of oxygenases mainly involved in biodegradation of organic molecules. The oxygenases having anabolic properties have also been included in this database.

## Construction

### Database design and development

**Figure 3:**
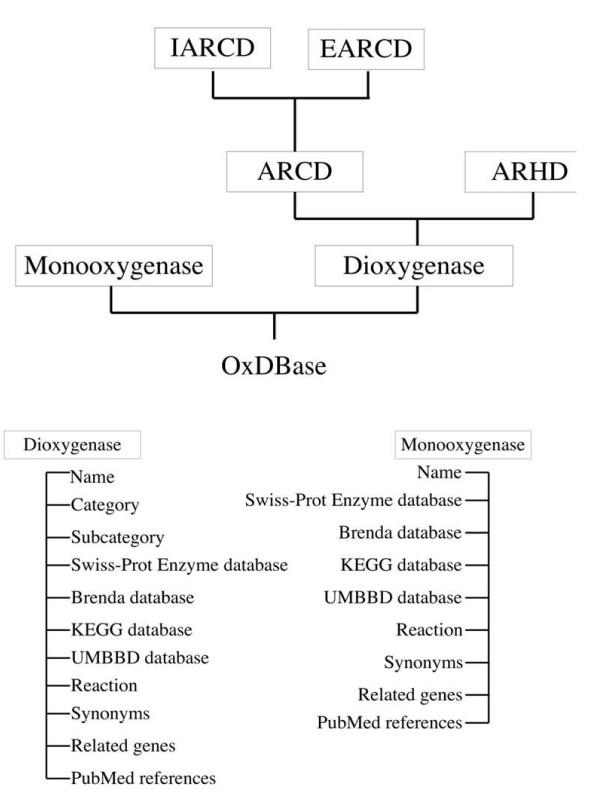
**Overall architecture of OxDBase**.

The PostgreSQL relational database management system (RDBMS) is the main work-horse of OxDBase. It has been used for storing, retrieval and managing the data. The scripts, which provide interface between user and database, were written in PERL and CGIPerl. For accessing information from PostgreSQL Pgperl has been used. The server OxDBase has been developed and launched on SUN solaris 10.0 environment on T1000 machine using Apache sever. Database entries were collected from different sources such as the published literature like PubMed , different existing databases such as UM-BBD , KEGG , ENZYME , BRENDA . The overall architecture of OxDBase is shown in Fig. 3. The database contains two tables housing information of 118 monooxygenases and 119 dioxygenases respectively.

### Data content and scope

OxDBase is a comprehensive database to provide information about oxygenases (both mono- and di-oxygenase) compiled from published literature and databases. The information about each entry includes: i) name and chemical structure of substrate and product; ii) link to the gene or protein sequence using NCBI database; iii) link to related PDB structures in the Protein Data Bank; iv) link to the key external databases such as SWISS-PROT ENZYME, BRENDA, KEGG and UM-BBD databases (wherever possible, the International Union of Biochemistry and Molecular Biology (IUBMB) name along with different synonyms by which that enzyme is known); and v) link to the related published literature at PubMed journals database has also been provided (Table 1). All entries of the database are assigned a unique accession number to identify them unambiguously.

**Table 1:** Description and content of fields associated with each entry of OxDBase database.

**Entry Field**	**Description**
Name	Wherever possible IUBMB Name or common name of the enzyme
Category	Chemical nature of substrate of dioxygenase
Subcategory	Specificity and nature of oxidation of dioxygenase
Enzyme database	The Enzyme Commissions identification (EC) number, link to the SWISS PROT ENZYME database
BRENDA database	The EC number and link to the BRENDA Enzyme information system
KEGG database	Link to molecular interaction network of Kyoto Encyclopedia of Genes and Genomes (KEGG) along with EC number
UMBBD database	The accession number of University of Minnesota Biocatalysis/biodegradation database
Reaction	The name and chemical structure of substrate and product
Synonyms	Popular names other than the IUBMB
Related genes	Link to the NCBI Entrez-gene database
Related structures	PDB four letter identification code plus a link to the corresponding information
PubMed reference	Link to the relevant literature to the PubMed database

### Categorization and classification of data

All entries of OxDBase are divided into two broad classes i.e. monooxygenases and dioxygenases depending on the number of atomic oxygen used during oxidation. On the basis of their mode of action dioxygenases are further categorized into aromatic ring cleavage dioxygenase (ARCD) and aromatic ring hydroxylating dioxygenase (ARHD) [12]. Depending upon the position of ring cleavage with respect to the hydroxyl groups, ARCDs are again divided into intradiol aromatic ring cleaving dioxygenase (IARCD) and extradiol aromatic ring cleavage dioxygenase (EARCD).

### Searching the database

OxDBase provides a number of methods to search the database. The following are the major ways: (i) generalized search using keywords to search in all fields of database; (ii) The Enzyme Commission number (EC number) based searching that allows extraction of a unique OxDBase entry; and (iii) class based searching which restricts the search within a specified class (described in categorization and classification of enzymes).

**Figure 4:**
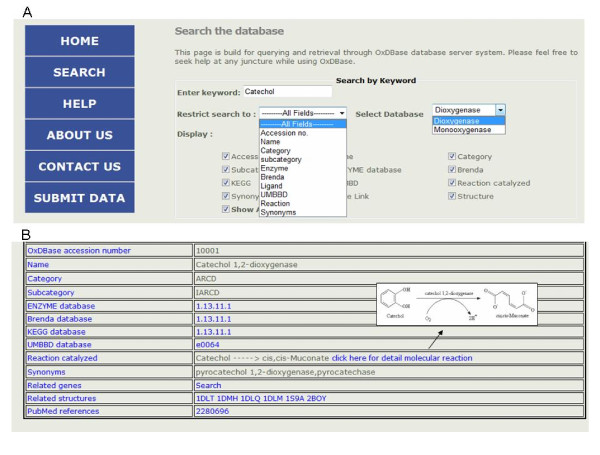
**Overview of the OxDBase search A) for keyword search; and B) output of keyword search**.

In short, keyword search allows users to data mine on all fields of the database ('EC number', 'IUBMB as well as other popular names', 'Publication Reference', 'Reactant and Substrate'). The keyword search could also be restricted to a particular field and it also allows users to select the fields to be displayed. An example of keyword search is shown in Fig. 4A, where keyword 'catechol' is searched in any field of database. The output/result of this keyword search is shown in Fig. 4B.

## Potential utility and limitations

**Figure 5:**
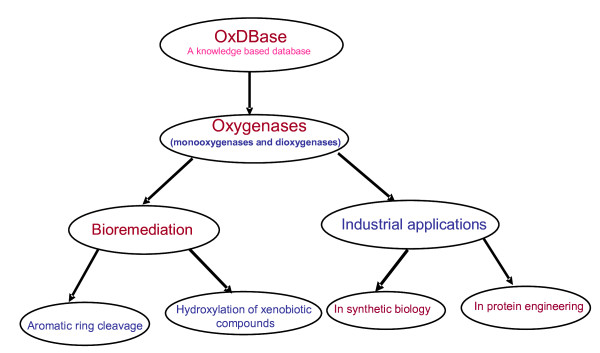
**Potential uses of oxygenases**.

OxDBase is a knowledge based database that provides comprehensive information about oxygenases including both monooxygenases and dioxygenases. The mechanism of action of the oxygenases is based on hydroxylation of the target molecule. During the recent years, selective hydroxylation of aromatic ring has gained attention in the synthetic biology because of the use of hydroxylated aromatics as drug intermediates. For example, the large scale industrial production of carticosterone, cis-cis muconic acid, pravastatin, indigo and 4-hydroxyproline have been achieved by hydroxylation mechanism of oxygenases [13]. Therefore, the information provided by OxDBase, particularly reaction catalyzed by oxygenases would be a very useful tool for synthesis of various biologically active compounds. OxDBase also provides information of the genes and three dimensional structure of the oxygenases which can help in site directed mutagenesis of the enzymes to improve their catalytic properties. The entries of the oxygenases in OxDBase are linked to various existing database to provide detailed information of oxygenases. Since oxygenases-catalyzed biotransformations of the toxic xenobiotic compounds help in reducing the toxicity of the xenobiotics, therefore, detailed information of these oxygenases would increase our understanding of biodegradation process. The potential uses of these oxygenases have been shown in fig. 5. We hope the OxDBase would be very useful tool for development of better bioremediation strategies as well as synthesis of biologically active compounds.

At present, OxDBase has 237 entries of distinct oxygenases. Among them, 118 belong to monooxygenases and 119 related to dioxygenases. The primary aim of OxDBase is to provide detailed information of all known oxygenases because of their wide use in synthetic chemistry and bioremediation. Hence, inspite of the limited information available about oxygenases, OxDBase is largely complete and of considerable importance. As new data becomes available, the database will also increase in size.

## Submission and updation of OxDBase

**Figure 6:**
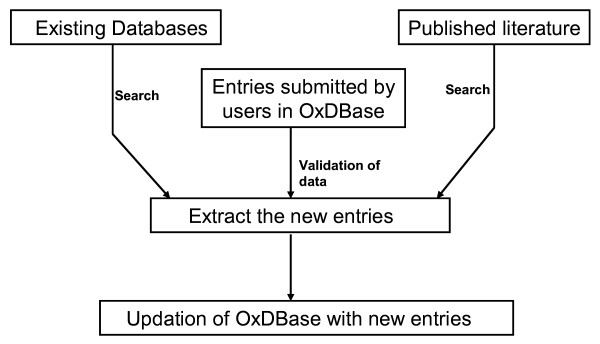
**A flow chart showing the mechanism for the curation and updation of the database**.

The web server allows user to submit new entry of oxygenase on-line by filling a HTML form. However, before including in OxDBase we will confirm the validity of new entry in order to maintain the quality. Our team is also searching and adding new entries of oxygenases in database from published literature. The mechanism followed for the curation and updation of the database has been shown in fig. 6. In order to maintain the consistency we will revive the OxDBase database quarterly.

## Conclusion

OxDBase is a unique database which provides comprehensive information about oxygenases. It is a platform from which users can easily retrieve information on all available oxygenases. The present database would increase our understanding of the biological, biochemical, genomic, evolutionary and structural properties of oxygenases that could be exploited for industrial and bioremediation applications.

## Future work

As regards to the future work the database needs to be maintained and developed further, ensuring the links to all external databases remain correct and newly published data is added. We hope, over the time, database size will increase with accumulation of more experimental information. In addition we also hope that data compilation and distribution through a publicly available medium will help in biodegradation research.

## Availability and requirements

OxDBase is freely available at .

## List of abbreviations used

FAD: Flavin Adenine Dinucleotide; NADH: Nicotinamide Adenine Dinucleotide Reduced; NADP: Nicotinamide Adenine Dinucleotide Phosphate Reduced; PERL: Practical Extraction and Report Language; NCBI: National Center for Biotechnology Information; PDB: Protein Data Bank.; BRENDA: The Comprehensive Enzyme Information System; UM-BBD: University of Minnesota Biocatalysis/Biodegradation Database; IUBMB: International Union of Biochemistry and Molecular Biology; KEGG: Kyoto Encyclopedia of Genes and Genomes; ENZYME: Enzyme Nomenclature Database.

## Competing interests

The authors declare that they have no competing interests.

## Authors' contributions

PKA and AC collected the data from literature and existing databases. MK and PKA created tables in PostgreSQL and developed web server. RKJ and GPSR conceived the project, coordinated it and refined the manuscript drafted by PKA and AC. All authors have read and approved the final manuscript.
